# Study on Binocular Accommodative Function in Children With Different Types of Intermittent Exotropia

**DOI:** 10.3389/fped.2021.726013

**Published:** 2021-10-18

**Authors:** Dan Li, Kun-Ling Li, Jing Wang, Lin-Lin Du, Li Li

**Affiliations:** ^1^Department of Ophthalmology, Affiliated Hospital of Hebei University, Baoding, China; ^2^Department of Pediatrics, Central Blood Station of Baoding, Baoding, China

**Keywords:** intermittent exotropia, accommodative amplitude, accommodative facility, monocular, binocular

## Abstract

**Objective:** This study aims to investigate the monocular and binocular accommodative amplitude (AMP) and accommodative function (AF) in children with different types of intermittent exotropia (IXT).

**Methods:** A total of 40 children with IXT were enrolled in the study. Monocular and binocular AMP and AF were measured using the modified approach method and the ±2D flip method, and the differences between the fixing and non-fixing eyes of non-strabismic children and children with different types of IXT were compared.

**Results:** The AMP of the fixing eyes of children with IXT was lower than that of their non-fixing eyes (*p* = 0.007). Conversely, the AF was higher in the fixing eyes than in the non-fixing eyes (*p* < 0.001). The AMPs of each group of children with IXT were lower than those of the control group, while the AMP of the group with convergence insufficiency was lower than that of the other two groups with IXT. In addition, the AF of the group with convergence insufficiency was lower than that of the group with basic exotropia and the control group (*p* < 0.05).

**Conclusion:** There is a difference in accommodation between the fixing and non-fixing eyes of children with IXT, and the degree of variation depends on the type of IXT. Moreover, the binocular accommodative function of children with IXT is lower than that of non-strabismic children.

## Introduction

Exotropia is the deflection of the optic axis caused by poor binocular fusion function, whereby the optic axis is unable to control the anteroposterior position. Its pathogenic factors include hereditary disease, prenatal adverse environments (such as maternal toxicological exposure), preterm labor, perinatal disease, and a family history of strabismus, astigmatism, and anisometropia. A 2007 paper by Chia also considers myopia to be one of the risk factors for exotropia (1).

Intermittent exotropia (IXT) is a common form of exotropia, accounting for 50–90% of all cases (2). This disorder usually occurs in children and has been studied globally by ophthalmologists (3). The onset of IXT is mainly caused by an imbalance in the convergence and abduction functions. Accommodation and convergence can be linked processes: patients with IXT may increase accommodative convergence to maintain monocular vision in both eyes (4).

At present, Burian's classification of IXT identifies four types of the condition based on the differences between distance and near deviation (5): (1) basic exotropia, where the near and distance deviations are almost equal; (2) convergence insufficiency exotropia, where the deviation of myopia is demonstrably greater than that of hyperopia (≥15^Δ^); (3) divergence excess exotropia, where the deviation of hyperopia is greater than that of myopia (≥15^Δ^); and (4) false divergence excess exotropia, where the deviation of hyperopia appears greater than that of myopia, but when both eyes wear +3D spherical lenses or one eye is covered for 1 h, the deviation of hyperopia and myopia is found to be more or less the same.

Studies of IXT traditionally focus on surgical methods and outcomes, eye position control, and stereopsis (6). Recently, the focus has shifted to include the visual study of patients with IXT and abnormal accommodation. However, the current research is insufficient (7). Although recent studies show that the difference in accommodative amplitude (AMP) and accommodative function (AF) between the fixing and non-fixing eyes in IXT patients is significant (8), little research has been conducted in this area or on the subject of AF in different types of IXT. Therefore, in this study, we attempt to establish the characteristics of binocular accommodative function in children with IXT by using retrospective analysis to analyze and observe the AF parameters of the fixing and non-fixing eyes. We also compare the differences in binocular accommodation across the various types of IXT.

## Methods

### Study Subjects

A total of 40 children (19 boys and 21 girls) with IXT, treated as outpatients at our hospital between December 2018 and June 2020, were enrolled in the study. All were between 6 and 12 years of age. The research process and aims of the study were explained to the children's parents, and their consent to participate was obtained. The study was reviewed and approved by the ethics committee of our hospital (number HDFYLL-2020-068).

Patients were included in the study if they met the following criteria: exotropia deviation ≥15Δ, ability to control the anteroposterior position, best-corrected visual acuity (BCVA) ≥0.8, and normal eye movement. Patients with the following conditions were excluded from the study: >1D anisometropia and astigmatism, amblyopia, nystagmus, vertical strabismus, AV syndrome, dissociated strabismus, or those undergoing accommodative training or with a history of strabismus surgery. Subjects underwent routine ophthalmic examinations to exclude anterior and posterior segmental organic diseases and to ensure no systemic diseases were present that could affect their cooperation in the study.

### Grouping Standard for the Subjects

The children with IXT were split into three groups based on the differences in 33 cm and 6 m exotropia deviation in triangular prism inspection after one eye was covered for an hour. Group 1 included 12 children with convergence insufficiency [where 33 cm exotropia deviation was significantly greater than 6 m exotropia deviation (≥15Δ)]. Group 2 included 18 children with basic exotropia (where 33 cm exotropia deviation was equal to 6 m exotropia deviation). Group 3 included 10 children with divergence excess exotropia [where 6 m exotropia deviation was significantly greater than 33 cm exotropia deviation (≥15Δ)]. Group 4, the control group, was made up of 20 non-strabismic children.

### Routine Examination

All patients underwent an eye test. Those with ametropia received mydriatic optometry following ciliary muscle paralysis. Their pupils were then re-examined after recovery, and the mean refractive spherical equivalent was recorded. The assessments were carried out on the basis of correction of refraction, and both eyes were examined in the order of fixing eye then non-fixing eye. Each measurement was recorded three times, and a mean value was taken.

To determine IXT of the fixing eye, both eyes were repeatedly covered and uncovered during the examination. The fixing eye was defined when the eye was fixed, and the non-fixing eye was defined as the eye that moved outward from the center after removing the cover.

AMP measurements were obtained using a modified approach method (9). The subjects were asked to look at a near visual target 40 cm away from the eye. A negative lens (−4.0 D) was added in front of the eye to make the near point seem further away. The visual target was slowly moved closer to the subject until they reported blurring, and the distance at this point (the near-point distance) was recorded. The close-point distance was then calculated using the formula AMP = 100/near-point distance (in cm). The final AMP value was obtained by adding 4.0 D.

AF was determined using the ±2 D flip method. Again, the subjects were required to look at a near visual target 40 cm away from the eye. A flip lens was added in front of the eye, and the subjects were asked to report when the visual target became clear following each flip. The lens was flipped from −2.00 D to +2.00 D and then back again, and the number of cycles per minute was recorded.

### Statistical Analysis

The measurement data were represented by mean ± standard deviation ($\bar{x}$ ± s) and analyzed using SPSS 17.0 software. The data met normal distribution in a normality test. The AMP and AF of the fixing and non-fixing eyes in children with IXT were analyzed using a paired *t*-test. One-way analysis of variance was then adopted for the general data and the binocular AMP and AF of the four groups. Further pairwise comparison was carried out for statistically significant values.

## Results

### General Information

As shown in Table 1, the mean age of the children with convergence insufficiency was 9.08 ± 1.73; with basic exotropia, 9.38 ± 1.91; with divergence excess exotropia, 9.70 ± 2.05. The mean age of the control group was 9.80 ± 1.90. The difference in age between the groups was not statistically significant (*p* > 0.05). The right and left eye diopters of the four groups were also compared. Again, the difference was not statistically significant (*p* > 0.05).

**Table 1:** Comparison of general information among four groups ($\bar{\text{x}}$ ± *s*).

**Groups**	**Case*s* (*n*)**	**Age (years)**	**Fixing eyes diopter**	**Non-fixing eye diopter**	**Refractive error**
Convergence insufficiency group	12	9.1 ± 1.7	−1.15 ± 0.94	−0.98 ± 0.79	−0.17 ± 0.43
Basic group	18	9.4 ± 1.9	−0.93 ± 0.84	−0.83 ± 0.20	0.08 ± 0.33
Divergence excess group	10	9.7 ± 2.1	−1.27 ± 1.32	−1.33 ± 1.34	0.05 ± 0.28
Control group	20	9.8 ± 1.9	−1.13 ± 1.04	−1.25 ± 1.19	0.02 ± 0.40
*F*		0.375	0.363	0.658	0.723
*p*		0.771	0.78	0.581	0.543

Further pairwise comparisons reveal that exotropia deviation in children with convergence insufficiency was less than in the group with divergence excess exotropia (*p* = 0.002). The difference in 6 m and 33 cm exotropia deviation was not statistically significant (*p* > 0.05) between the other groups (Table 2).

**Table 2:** Comparison of far and near exotropia deviation of intermittent exotropia among three groups ($\bar{\text{x}}$ ± *s*).

**Groups**	**33 cm exotropia deviation (PD)**	**6 cm exotropia deviation (PD)**
Convergence insufficiency group	−41.67 ± 11.55	−25.83 ± 11.04
Basic group	−35.83 ± 15.36	−35.28 ± 13.98
Divergence excess group	−28.50 ± 12.48	−45.00 ± 13.54
*F*	2.543	5.885
*p*	0.09	0.006

### Comparison of the AMP and AF of the Fixing Eyes and Non-fixing Eyes in Children With IXT

Table 3 shows that the AMP of the fixing eyes of children with IXT was lower than that of their non-fixing eyes. The difference was statistically significant (*p* = 0.007). However, the AF of their fixing eyes was higher than that of their non-fixing eyes. This difference was also statistically significant (*p* < 0.001).

**Table 3:** Comparison of AMP and AF between fixing eyes and non-fixing eyes in IXT children ($\bar{\text{x}}$ ± *s*).

**Eyes**	**Number of eyes (*n*)**	**AMP (*D*)**	**AF (times/min)**
Fixing eyes	40	13.46 ± 1.34	11.00 ± 1.95
Non-fixing eyes	40	13.90 ± 1.41	9.61 ± 2.10
*t*		−2.85	7.486
*p*		0.007	<0.001

### Comparison of Binocular AMP and AF Among the Four Groups of Children

The binocular AMP and AF of the four groups were compared. Table 4 shows that the difference was statistically significant (*p* < 0.05). An additional pairwise comparison indicates that the AMP of each group of children with IXT was lower than that of the control group (*p* < 0.05). The AMP of the group with convergence insufficiency was lower than that of the other groups with IXT (*p* < 0.05). The AF of the group with convergence insufficiency was also lower than that of the group with basic exotropia and the control group (*p* < 0.05). This difference was not statistically significant in the other groups (*p* > 0.05; see Table 5).

**Table 4:** Comparison of binocular AMP and AF among four groups ($\bar{\text{x}}$ ± *s*).

**Groups**	**AMP (*D*)**	**AF (times/min)**
Convergence insufficiency group	8.42 ± 1.16	8.17 ± 1.27
Basic group	9.89 ± 1.71	9.67 ± 1.85
Divergence excess group	10.10 ± 1.66	9.30 ± 2.16
Control group	12.73 ± 1.3	10.67 ± 1.76
*F*	19.615	4.489
*p*	<0.001	0.007

**Table 5:** Pairwise comparison of binocular AMP and AF among four groups.

**Groups**	**AMP (D)**	**AF (times/min)**
Convergence insufficiency group and basic group	0.012^*^	0.028^*^
Convergence insufficiency group and divergence excess group	0.012^*^	0.143
Convergence insufficiency group and control group	<0.001^*^	0.001^*^
Basic group and divergence excess group	0.725	0.603
Basic group and control group	<0.001^*^	0.114
Divergence excess group and control group	<0.001^*^	0.065

* *Means that p < 0.05 refers to statistical significance*.

## Discussion

In recent years, awareness of IXT pathogenesis has developed, and it has become understood that the accommodation and convergence functions are closely related to the occurrence and development of the condition. Studies of differences in binocular accommodation in patients with IXT have shown that the difference in the monocular AMP and AF of fixing and non-fixing eyes is significant and that the AF of non-fixing eyes is lower than that of fixing eyes (10, 11). These results are supported by the findings of this study, where it was observed that the difference in the monocular AMP and AF of fixing eyes and non-fixing eyes was statistically significant (*p* < 0.001) and that the AF of non-fixing eyes was 9.61 ± 2.10 times/min, which was significantly lower than the 11.00 ± 1.95 times/min of fixing eyes.

AMP refers to the maximum near accommodative scope that the eye can reach. It is measured using the clinical approach method. AF refers to the relaxation and utilization accommodation capacity of the eye. Within 1 min, both eyes of children aged 8–12 with no pre-existing conditions should see no less than five cycles, while the single eye should see at least seven cycles. In patients with IXT, a non-fixing eye typically exhibits deviation. However, to maintain eye position, the non-fixing eye may use convergence. This will lead to a difference in binocular accommodation, resulting in bilateral eye competition, which will cause a decrease in AF and accommodative response (12). This is consistent with the results of this study, in which it was found that the AMP of non-fixing eyes in patients with IXT is higher than that of their fixing eyes, suggesting that their fixing eyes should exhibit clear vision in everyday use.

The non-fixing eye, however, commonly manifests itself as a squint. To maintain the eye position in a highly accommodative state for an extended period of time, the eyes require a close-up view; this is especially true in school-age children. Therefore, they contribute to greater accommodative convergence, showing AMP magnification in the visual function examination. However, previous studies have demonstrated conflicting results. For example, some observed myopic anisometropia and found that the accommodative strength of non-fixing eyes was lower than that of fixing eyes, and also that the myopic diopter of non-fixing eyes was significantly higher than that of fixing eyes (13). In the present study, the difference in binocular refractive spherical equivalent between fixing and non-fixing eyes was not statistically significant (*p* = 0.543); whether the overlapping effect of IXT and refractive factors are the reasons for this difference requires further investigation.

The present study found that both the binocular AMP and AF of children with IXT were significantly lower than those of the non-strabismic children in the control group. This may be because the accommodative response of binocular fixation is higher than that of monocular fixation. It is suggested that the level of linkage sensitivity between the two eye movement systems, accommodation and convergence, is low. Typically, the accommodation of both eyes is coordinated and unified. However, patients with IXT do not have the same accommodative response in the dominant and non-dominant eye when both eyes are fixed on the near visual target. Patients with exotropia are in a state of accommodative tension, and it is difficult for their eyes to relax when the convergence remains fixed. Therefore, the transit time may be extended by passing the positive lens in the AF examination. Patients with exotropia need to mobilize more convergence functions during binocular fixation than those with healthy eyes to achieve binocular haplopia. Accordingly, the increased convergence will result in greater accommodation (14). Some studies have found that the binocular AF of patients with IXT is significantly lower than that of a control group, which is consistent with the results of this study (15).

The present study also examined the accommodative parameters of patients with different types of IXT, finding the lowest AMP (8.42 ± 1.16 D) in the group of children with convergence insufficiency. This difference was statistically significant compared with the other groups. The highest AMP (10.10 ± 1.66 D) was found in children with divergence excess exotropia, and the difference was not statistically significant when compared with children with basic exotropia. The lowest AF (8.17 ± 1.27 times/min) was also found in children with convergence insufficiency, while the highest AF (9.67 ± 1.85 times/min) was found in those with basic exotropia. This difference was again found to be statistically significant. However, the difference was not significant when compared with children with divergence excess exotropia. These results indicate that children with convergence insufficiency have a lower accommodative function, which may require further medical treatment.

Convergence insufficiency accounts for 19.5% of IXT cases (16). These patients are characterized by a low accommodative convergence/accommodation ratio and a decrease in positive fusional convergence when viewing a near object. Once the near point of convergence falls below normal distance (10 cm), symptoms (such as visual fatigue, dyslexia, and blurred near vision) emerge. The success rate of surgery is low. Compared with basic exotropia and divergence excess exotropia, the control of eye position in convergence insufficiency IXT is relatively weak. Patients with convergence insufficiency are also more likely to suffer from anisometropia than patients with other types of IXT, and the difference in binocular AF is even greater. As such, it is speculated that abnormal AF may be related to the abnormality of binocular visual function (17, 18). In this study, the difference in the binocular diopter spherical equivalent of children with convergence insufficiency was higher than that of the three other groups, but the difference was not statistically significant.

Refraction may affect accommodative function. In this study, there was no significant difference in the spherical equivalent diopter of the four groups. Therefore, the influence of refractive factors can be excluded. Accommodative problems increase the risk of myopia in young children with IXT (19), and myopia and IXT are considered important co-existing diseases (20). This is reflected in the overlapping and mutual promotion of the pathogenesis and progression of the two diseases. Unfortunately, this study's cross-sectional analysis was limited to the AMP and AF of children with IXT. Moreover, only a small number of cases were observed. Therefore, a larger sample size should be used for future investigations.

## Conclusion

There is a significant difference in accommodation between the fixing and non-fixing eyes of children with IXT and between the fixing and non-fixing eyes of children with different types of IXT. AF is lowest in children with convergence insufficiency and highest in those with divergence excess exotropia or basic exotropia. Furthermore, binocular AF in children with IXT is lower than that in non-strabismic children. However, this study only conducted cross-sectional investigations on AMP and AF in children with IXT, and the number of observed cases was small. Therefore, the sample size should be further expanded in future observations.

## Data Availability Statement

The raw data supporting the conclusions of this article will be made available by the authors, without undue reservation.

## Ethics Statement

The study was reviewed and approved by the Ethics Committee of the Affiliated Hospital of Hebei University (No.: HDFYLL-2020-068). Written informed consent to participate in this study was provided by the participants' legal guardian/next of kin.

## Author Contributions

K-LL and L-LD conceived the idea and conceptualized the study. L-LD and LL collected the data. DL and K-LL analyzed the data. DL and JW drafted the manuscript and reviewed the manuscript. All authors read and approved the final draft.

## Funding

This study was funded by the Hebei Baoding Science and Technology Plan Project (2041ZF173) and Hebei Medical Science Research Project (20210175). The funding body had no role in the design of the study and collection, analysis, and interpretation of data and in writing the manuscript.

## Conflict of Interest

The authors declare that the research was conducted in the absence of any commercial or financial relationships that could be construed as a potential conflict of interest.

## Publisher's Note

All claims expressed in this article are solely those of the authors and do not necessarily represent those of their affiliated organizations, or those of the publisher, the editors and the reviewers. Any product that may be evaluated in this article, or claim that may be made by its manufacturer, is not guaranteed or endorsed by the publisher.
